# Parsing propagule pressure: Number, not size, of introductions drives colonization success in a novel environment

**DOI:** 10.1002/ece3.4226

**Published:** 2018-07-20

**Authors:** Michael J. Koontz, Meagan F. Oldfather, Brett A. Melbourne, Ruth A. Hufbauer

**Affiliations:** ^1^ Graduate Group in Ecology University of California Davis California; ^2^ Graduate Degree Program in Ecology Colorado State University Fort Collins Colorado; ^3^ Department of Bioagricultural Science and Pest Management Colorado State University Fort Collins Colorado; ^4^ Department of Integrative Biology University of California Berkeley California; ^5^ Department of Ecology and Evolutionary Biology University of Colorado Boulder Colorado

**Keywords:** biocontrol, conservation, invasion, microcosm, population dynamics, propagule pressure, reintroduction, simulation, stochasticity

## Abstract

Predicting whether individuals will colonize a novel habitat is of fundamental ecological interest and is crucial to conservation efforts. A consistently supported predictor of colonization success is the number of individuals introduced, also called propagule pressure. Propagule pressure increases with the number of introductions and the number of individuals per introduction (the size of the introduction), but it is unresolved which process is a stronger driver of colonization success. Furthermore, their relative importance may depend upon the environment, with multiple introductions potentially enhancing colonization of fluctuating environments. To evaluate the relative importance of the number and size of introductions and its dependence upon environmental variability, we paired demographic simulations with a microcosm experiment. Using *Tribolium* flour beetles as a model system, we introduced a fixed number of individuals into replicated novel habitats of stable or fluctuating quality, varying the number of introductions through time and size of each introduction. We evaluated establishment probability and the size of extant populations through seven generations. We found that establishment probability generally increased with more, smaller introductions, but was not affected by biologically realistic fluctuations in environmental quality. Population size was not significantly affected by environmental variability in the simulations, but populations in the microcosms grew larger in a stable environment, especially with more introduction events. In general, the microcosm experiment yielded higher establishment probability and larger populations than the demographic simulations. We suggest that genetic mechanisms likely underlie these differences and thus deserve more attention in efforts to parse propagule pressure. Our results highlight the importance of preventing further introductions of undesirable species to invaded sites and suggest conservation efforts should focus on increasing the number of introductions or reintroductions of desirable species rather than increasing the size of those introduction events into harsh environments.

## 1 INTRODUCTION

Colonization is the ecologically fundamental process of population establishment in an unoccupied location, and it underlies the past, present, and future distributions of species. Colonization occurs naturally but is increasingly prevalent due to anthropogenic influences (Cassey, Blackburn, Duncan, & Chown, 2005; Ricciardi, 2007; Sakai et al., 2001). Incipient populations often face environments that are entirely novel, which is especially likely in the case of anthropogenic colonization (Cassey et al., 2005; Ricciardi, 2007). Regardless of whether colonization events to novel habitats are natural (e.g., range expansion) or human‐mediated (e.g., biological invasions, reintroductions of rare species, release of biological control agents), their successes or failures have significant implications for natural resource managers and society (Mack et al., 2000).

Most introductions to novel habitats fail, and colonization success can be difficult to predict (Lockwood, Cassey, & Blackburn, 2005; Zenni & Nuñez, 2013). Incipient populations are commonly small, and face threats from environmental, demographic, and genetic stochasticity (Fauvergue, Vercken, Malausa, & Hufbauer, 2012; Lande, 1988, 1993). Furthermore, the success of any given population can be idiosyncratic with respect to taxonomy and geography (Lockwood et al., 2005; Lodge, 1993). Thus, it is crucial to understand more general features of the colonization process beyond the particular invading organism or the particular invaded environment (Lockwood et al., 2005). Propagule pressure is one such general feature that is a consistent predictor of colonization success in novel habitats (Colautti, Grigorovich, & MacIsaac, 2006; Jeschke, 2014; Lockwood et al., 2005; Simberloff, 2009).

Propagule pressure is the total number of potentially reproductive individuals (e.g*.,* adults, eggs, seeds, vegetative material) introduced to an area (Novak, 2007). It is often described in this broad sense, which belies its complexity. Two important components of propagule pressure are the number of introduction events— sometimes termed propagule number, and the average number of individuals per introduction event— sometimes termed propagule size (sensu Fauvergue et al., 2012). Here, we use “introduction regime” to refer to different combinations of the number of introduction events and the number of individuals introduced per event. The same total propagule pressure, *N*, is realized by different introduction regimes depending on how those *N* individuals are distributed in time or space (Haccou & Iwasa, 1996). An introduction regime of *N* individuals lies on a continuum bounded by maximizing the number of individuals introduced per event (all *N* individuals introduced in one event to the same location) and maximizing the number of introduction events (one individual introduced in each of *N* sequential events through time or to each of *N* unique locations).

Colonization success increases with total propagule pressure, but it is unclear whether the correlation is driven by the number of individuals introduced per event or the number of introduction events (Colautti et al., 2006; Lockwood et al., 2005; Simberloff, 2009). Historical data suggest that multiple introductions can facilitate population establishment, but conclusions from these studies are limited by the inability to control for the total number of individuals introduced (Blackburn & Duncan, 2001; Fauvergue et al., 2012; Grevstad, Coombs, & McEvoy, 2011; Hopper & Roush, 1993). Models that hold the total propagule pressure constant agree that multiple, small introductions distributed across space will lead to greater establishment probability compared to a single, large introduction when Allee effects are weak (Grevstad, 1999; Haccou & Iwasa, 1996; Schreiber & Lloyd‐Smith, 2009). However, both modeling and empirical approaches have generated conflicting views on how an introduction regime affects colonization success when introductions are distributed through time, a situation in which individuals from later introductions interact both demographically and genetically with individuals from previous introductions.

There is evidence from both models and experiments that colonization success can increase with more, smaller introduction events through time. Branching process models show that, in the long run, several small introductions will be more likely to successfully establish a population than a single large introduction (Haccou & Iwasa, 1996; Haccou & Vatutin, 2003). This finding was corroborated by simulations with no Allee effects (Drolet & Locke, 2016). In a *Daphnia* microcosm experiment, increasing introduction frequency (proportional to the number of introductions) positively affected population growth, but the number of individuals per introduction had no detectable effect (Drake, Baggenstos, & Lodge, 2005). Establishment probability was only affected by total propagule pressure and did not increase with increasing introduction frequency (Drake et al., 2005). However, Drake et al. (2005) did not continue scheduled introductions if a population went extinct, denying those populations one of the main benefits of repeated introductions and creating variability in the total propagule pressure. In a more recent experiment, several small introductions through time led to a 65% increase in abundance of successfully colonizing invasive Pacific Oyster (*Crassostrea gigas*) compared to a single large introduction (Hedge, O'Connor, & Johnston, 2012).

There is also evidence from both models and experiments that colonization success can be greater with fewer larger introduction events than with multiple smaller introductions through time. For instance, in simulations of bird invasions by Cassey, Prowse, and Blackburn (2014), a single large introduction event always led to the greatest establishment probability. Drolet and Locke (2016) highlighted a key role of positive density dependence in favoring fewer larger introductions; when they included Allee effects in their simulations, colonization success was enhanced with fewer larger introductions—the reverse of their observed pattern without Allee effects. In a field experiment with the psyllid biocontrol agent, *Arytainilla spartiophila*, the number of individuals per introduction event was a better predictor of establishment success than the number of introduction events (Memmott, Craze, Harman, Syrett, & Fowler, 2005). In this case, however, the introduction regimes with the most individuals per event also had the highest total propagule pressure (Memmott et al., 2005). In an experiment that controlled total propagule pressure, a single, large introduction of the nonnative mysid, *Hemimysis anomala*, led to larger populations and greater survival probabilities compared to several, small introductions through time (Sinclair & Arnott, 2016).

Environmental stochasticity in the recipient environment may also affect which introduction regime is optimal for colonization. Branching process models show that a more variable environment reduces the probability of population establishment for all introduction regimes (Haccou & Iwasa, 1996; Haccou & Vatutin, 2003), and simulations of introductions distributed in space suggest that greater environmental variability will magnify the benefit of multiple introductions (Grevstad, 1999). However, simulations of introductions through time by Cassey et al. (2014) did not support either of these outcomes— a single, large introduction was most likely to establish a population even with extreme levels of environmental stochasticity.

Thus, modeling and empirical approaches have not resolved how different introduction regimes with a fixed total propagule pressure will affect colonization success when introductions are distributed through time. Furthermore, there has been no experimental test of whether variability in the recipient environment interacts with the introduction regime to affect colonization. We paired demographic simulations with a laboratory microcosm experiment using the red flour beetle, *Tribolium castaneum,* to reconcile conflicts in the literature and test how different introduction regimes implemented through time affect colonization success in novel habitats. We explicitly manipulated whether the novel habitat was stable or randomly fluctuating in quality to assess how the success of different introduction regimes may depend upon variability in the recipient environment. With the total number of individuals introduced held constant at 20, we varied the size and number of introduction events used to distribute those individuals in four different introduction regimes. We evaluated establishment probability and population size over seven discrete generations to ask: (a) does colonization success increase with more introduction events or with more individuals per introduction event?, and (b) does the effect of the introduction regime on colonization success depend on whether the recipient novel environment is stable or fluctuating through time?

## 2 METHODS

### 2.1 General framework

In simulations and a microcosm experiment, we evaluated the outcome of introducing 20 total individuals to one of two environmental contexts (a stable or fluctuating novel environment), varying the number of introduction events used to distribute those individuals through time. This total propagule pressure was low enough to allow some population extinction within an observable timeframe, but high enough to be representative of documented introductions in the literature (Berggren, 2001; Drake et al., 2005; Grevstad, 1999; Simberloff, 1989, 2009; Taylor, Jamieson, & Armstrong, 2005). The introduction regimes were: 20 individuals introduced in the first generation, 10 individuals introduced in each of the first two generations, five individuals introduced in each of the first four generations, and four individuals introduced in each of the first five generations. To create the fluctuating environment, we imposed a magnitude of variability corresponding to environmental stochasticity in nature, which leads to frequent, mild‐to‐moderate perturbations in population growth rate due to external forces (Lande, 1993). Populations were tracked for seven generations following the initial introduction.

Establishment probability and the size of established populations were used as measures of colonization success. Populations were deemed “established” for any time step in which they were extant and population size was noted for all extant populations in every time step. Establishment probability and mean population size were assessed in generation 7. We also assessed establishment probability and population size 3 generations after the final introduction event (*i.e.,* by assessing establishment and population size for the 20 × 1 regime at generation 3, 10 × 2 regime at generation 4, 4 × 5 regime at generation 6, and 5 × 4 regime at generation 7). By evaluating characteristics of these introduction scenarios at both an absolute time point (*e.g*., generation 7) and a relative time point (*e.g*., three generations after final introduction), we can account for how different biological phenomena may be important across different time frames of interest. For instance, assessment of colonization at a fixed time point may better capture the effect of environmental variation that begins with the first introduction regardless of introduction regime, or it may be more valuable in a conservation setting when metrics of success are the most meaningful after implementation of a management plan for a set amount of time. Alternatively, an assessment of colonization after a relative time point may better capture the influence of stochastic extinction or expression of genetic load, both of which grow more likely with each passing generation beyond the final introduction.

### 2.2 Study system

Our simulations and microcosm experiment use *Tribolium castaneum* (red flour beetles) to model the life history of organisms with discrete, nonoverlapping generations (e.g., annual insects and fishes) following Melbourne and Hastings (2008). Individual simulated colonists were randomly sourced from a randomly mating, infinitely large population. Individual experimental colonists came from a thoroughly mixed source population maintained at 800 individuals in each of the four temporal blocks over which the experiment was replicated. The four source populations were themselves sourced from the “SF” laboratory colony which was collected in the wild and maintained at thousands of individuals for ~15 generations prior to use in this experiment (Hufbauer et al., 2015). To obtain colonists, beetles from the source population were mixed freely on a plate, selected from many sections of the plate, and introduced to subsets of the experiment populations in a random order (about 24 subsets per block). All migrant females were assumed to arrive mated from the source population. The strong maternal effects exhibited by *Tribolium* flour beetles were reduced by rearing individuals from the source population on novel growth medium for one generation prior to using them as colonists (Hufbauer et al., 2015; Van Allen & Rudolf, 2013).

### 2.3 Simulations

Population dynamics were simulated with a Negative Binomial‐Binomial gamma (NBBg) model, which mechanistically describes change in population size in each discrete generation as a birth‐death process with cannibalism‐induced density dependence and four distinct forms of stochasticity: environmental stochasticity, skewed sex ratio, demographic heterogeneity, and demographic stochasticity (Melbourne & Hastings, 2008). For each combination of the two introduction regimes and two recipient environment types (described in “General Framework” above), we simulated 500,000 replicate populations for seven generations. The NBBg model captures the stochastic and deterministic ecological dynamics of the *Tribolium* system well, but does not include evolutionary processes (Hufbauer et al., 2015; Melbourne & Hastings, 2008).

The core of the NBBg model is a Ricker function (Equation (1)), where the mean size of population *i* at time *t* + 1 is a function of *F*_mated(*t*,*i*)_ (the number of mated females in population *i* at time *t*), *p* (the probability of an individual being female, 0.5), *R*_*t*,*i*_ (the density‐independent population growth rate for population *i* at time *t*), α (the egg cannibalism rate), and *N*_*t*,*i*_ (the total size of population *i* at time *t* representing the sum of the residents and the migrants).

(1) $$\mu_{t+1,i}\;=\;\frac{F_{{\mathrm{mated}}_{\left(t,i\right)}}}{p}R_{t,i}e^{-\alpha N_{t,i}}$$

The expected equilibrium population size for the Ricker model, when *N*_*t*+1_ is expected to be equal to *N*_*t*_, is:

(2) $$K\;=\;\frac{\log(R_{0})}{\alpha }$$

Environmental stochasticity is treated as variability in the potential density‐independent population growth rate arising from different unmeasured environmental conditions, and we model it (Equation (3)) by drawing the density‐independent population growth rate for population *i* at time *t* from a gamma distribution with a mean of *R*_0_ (the density‐independent population growth rate for the average environment) and shape parameter *k*_*E*_ (where a smaller value indicates greater environmental stochasticity).

(3) $$R_{t,i}∼\text{Gamma}\left(k_{E},\frac{R_{0}}{k_{E}}\right)$$

Stochasticity arising from a skewed sex ratio (Equation (4)) is modeled by drawing the number of resident and migrant females in the population from binomial distributions with a size of *N*_migrants(*t*,*i*)_ or *N*_residents(*t*,*i*)_ (the total number of migrants to or residents of population *i* at time *t*) and a probability of *p* (the probability of an individual being a female).

(4) $$\begin{array}{c} F_{{\mathrm{migrants}}_{\left(t,i\right)}}∼\text{Binom}\left(N_{{\mathrm{migrants}}_{{\left(t,i\right)}^{\prime }}}p\right)\\ F_{{\mathrm{residents}}_{\left(t,i\right)}}∼\text{Binom}\left(N_{{\mathrm{residents}}_{{\left(t,i\right)}^{\prime }}}p\right) \end{array}$$

Demographic heterogeneity is treated as variation in the expectation of the number of individuals in the next generation due to inherent differences in the egg‐laying capacity of mated females (*e.g*., different sized females may lay different average numbers of eggs). We model demographic heterogeneity (Equation (5)) by drawing the expectation of population size for population *i* at time *t *+ 1 from a gamma distribution with a mean of μ_*t*+1,*i*_ (the expected mean size of population *i* at time *t* + 1 derived from the Ricker function which only incorporates environmental stochasticity and skewed sex ratio) and a shape parameter *k*_*D*_ *F_mated(*t*,*i*)_ (where a smaller value of *k*_*D*_ indicates greater demographic heterogeneity, and the dependence of the shape parameter on *F*_mated(*t*,*i*)_ indicates greater demographic heterogeneity with fewer mated females at time *t*).

We model variation arising from demographic stochasticity (Equation (6)) by drawing the number of individuals in population *i* at time *t* + 1 from a Poisson distribution using the expectation of the population size at time *t* + 1 derived from Equation (5). Equations (5) and (6) represent a gamma mixture of Poissons, and so we simplify them to a negative binomial distribution (Equation (7)) with a mean of μ_*t*+1,*i*_ (derived from Equation (1)) and a size parameter of *k*_*D*_ *F_mated(*t*,*i*)_.

(5) $$E\left(N_{t+1,i}\right)∼\text{Gamma}\left(k_{D}F_{{\mathrm{mated}}_{{\left(t,i\right)}^{\prime }}}\frac{\mu_{t+1,i}}{k_{D}F_{{\mathrm{mated}}_{\left(t,i\right)}}}\right)$$

(6) $$N_{t+1,i}∼\text{Poisson}\left(E\left(N_{t+1,i}\right)\right)$$

(7) $$N_{t+1,i}∼\text{NegBinom}\left(\mu_{t+1,i},k_{D}F_{{\mathrm{mated}}_{\left(t,i\right)}}\right)$$

We assumed that females from the external source population arrived premated, so we imposed a mating function (Equation (8)) with two implications: (a) a population would deterministically go extinct if it comprised only nonmigrant females, and (b) in cases with an all‐female population and a mixture of residents and migrants, we only included the number of migrant females in the density‐dependent effect of the demographic heterogeneity term *k*_*D*_ *F_mated(*t*,*i*)_. This effect manifests via the number of eggs laid by females and only migrant females, being premated, would lay eggs.

(8) $$F_{{\mathrm{mated}}_{\left(t,i\right)}}\;=\;\left\{\begin{array}{c} F_{{\mathrm{migrants}}_{{\left(t,i\right)}^{\prime }}}\;\;\text{if}\;\;F_{{\mathrm{migrants}}_{\left(t,i\right)}}\;+\;F_{{\mathrm{residents}}_{\left(t,i\right)}}\;=\;N_{t,i}\\ F_{{\mathrm{migrants}}_{\left(t,i\right)}}\;+\;F_{{\mathrm{residents}}_{{\left(t,i\right)}^{\prime }}}\;\text{if}\;F_{{\mathrm{migrants}}_{\left(t,i\right)}}\;+\;F_{{\mathrm{residents}}_{\left(t,i\right)}}<N_{t,i} \end{array}\right.$$

To estimate the key parameters for the simulation model (α*, R*_0_ *, k*_*E*_, and *k*_*D*_), we censused 125 *Tribolium* populations one generation after establishing them at various, known densities (between 5 and 200 individuals) on the novel growth medium following the rearing procedure described in “Microcosm Experiment” below. We combined Equations (1), (3), (4), (7), and (8) into a single hierarchical model with weakly regularizing gamma priors taken from Melbourne and Hastings (2008) (Equation (9)) and fit the model to the population size data from the 125 populations in a Bayesian framework to generate posterior distributions for each of the parameters.

(9) $$\begin{array}{cc} & N_{t+1,i}∼\text{NegBinom}\left(\mu_{t+1,i},k_{D}F_{{\mathrm{mated}}_{\left(t,i\right)}}\right)\\ & \mu_{t+1,i}\;=\;\frac{F_{{\mathrm{mated}}_{\left(t,i\right)}}}{p}R_{t,i}e^{-\alpha N_{t,i}}\\ & R_{t,i}∼\text{Gamma}\left(k_{E},\frac{R_{0}}{K_{E}}\right)\\ & N_{t,i}\;=\;N_{{\mathrm{migrants}}_{\left(t,i\right)}}\;+\;N_{{\mathrm{residents}}_{\left(t,i\right)}}\\ & F_{{\mathrm{mated}}_{\left(t,i\right)}}\;=\;\left\{\begin{array}{c} F_{{\mathrm{migrants}}_{{\left(t,i\right)}^{\prime }}}\text{if}\;F_{{\mathrm{migrants}}_{\left(t,i\right)}}\;+\;F_{{\mathrm{residents}}_{\left(t,i\right)}}\;=\;N_{t,i}\\ F_{{\mathrm{migrants}}_{\left(t,i\right)}}\;+\;F_{{\mathrm{residents}}_{{\left(t,i\right)}^{\prime }}}\text{if}\;F_{{\mathrm{migrants}}_{\left(t,i\right)}}\;+\;F_{{\mathrm{residents}}_{\left(t,i\right)}}<N_{t,i} \end{array}\right.\\ & F_{{\mathrm{migrants}}_{\left(t,i\right)}}∼\text{Binom}(N_{{\mathrm{migrants}}_{{\left(t,i\right)}^{\prime }}}p)\\ & F_{{\mathrm{residents}}_{\left(t,i\right)}}∼\text{Binom}(N_{{\mathrm{residents}}_{{\left(t,i\right)}^{\prime }}}p)\\ & p\;=\;0.5\\ & R_{0}∼\text{Gamma}\left(2.6,1\right)\\ & \alpha ∼\text{Gamma}(0.0037,1)\\ & k_{E}∼\text{Gamma}\left(17.6,1\right)\\ & k_{D}∼\text{Gamma}\left(1.07,1\right) \end{array}$$

We fit the model using the nimble package in R (de Valpine et al., 2016) using a Metropolis‐Hastings random walk sampler with three chains having 50,000 samples each (including 10,000 samples that were removed for burn in). The chains converged (multivariate $\hat{R}$ for the four key parameters =1.01) and produced a sufficient number of effective samples (*R*_0_: 757, α: 548, *k*_*E*_: 4103, *k*_*D*_: 1703). All simulations using the posterior distributions of the parameters were performed in R (R Core Team 2017).

We used the posterior distribution of the estimated environmental stochasticity parameter, *k*_*E*_, for simulations in a stable environment because the 125 populations were reared on a single novel growth medium mixture (mixture 5, Supporting information Table S1). We parameterized a fluctuating environment simulation by increasing the standard deviation of the density‐independent per capita population growth rate by 10% compared to the stable environment for any given population at any given time step, a similar magnitude of variation as imposed by Cassey et al. (2014) (see Supporting information Methods S1). We used random sets of parameter values drawn from the samples of the posterior distributions of the key parameters to simulate the size of each population at time *t* + 1 given the size of that population at time *t*. By randomly drawing sets of values from the model‐estimated posterior distributions of parameters, we were able to propagate uncertainty in the model estimates of the parameters through the simulations and combine it with the deterministic and stochastic dynamics represented by the model itself.

### 2.4 Microcosm experiment

We founded 842 *Tribolium* populations with different introduction regimes (20 individuals in the first generation, 10 individuals in the first two generations, five individuals in the first four generations, or four individuals in the first five generations) and environments (stable or fluctuating) with between 96 and 120 replicate populations per treatment combination (Supporting information Table S3). Each population was reared in a 4 cm × 4 cm × 6 cm plastic box (AMAC Plastic Products) with two tablespoons (approximately 15 g) of freshly prepared growth medium that had been humidified for at least 24 hr. The growth medium used for the source population comprised 95% wheat flour (Pillsbury Co. or Gold Medal Products Co.) and 5% brewers' yeast (Sensient Flavors). We term this growth medium mixture the “natal medium” as it represents the natal environment of the colonists. Colonists were introduced into a novel growth medium comprising a small percentage of natal medium mixed with corn flour (Bob's Red Mill). All populations were reared in one of two dark incubators at 31° and approximately 70% relative humidity (standard conditions) and were haphazardly rotated between incubators weekly.

For each population in each generation, a known number of adults laid eggs for 24 hr in fresh medium and were then removed. Offspring were given 35 days to develop, and adults were then censused. Censused adults laid eggs on freshly prepared growth medium for 24 hr, completing their laboratory life cycle. We estimated the maximum observation error during census to be 4.6% (median: 0%, mean: 0.26%) with no detectable difference across observers or populations with different sizes.

Each replicate population experienced a novel environment that was either stable or randomly fluctuating through time. The same novel growth medium mixture containing 99.05% novel corn flour and 0.95% natal medium (mixture 5, Supporting information Table S1) was used for the stable environment for the duration of the experiment, which preliminary results indicated would yield a population growth rate of λ = 1.2 compared to a mean population growth rate of 3.36 on 100% natal medium. To create the fluctuating environment, we randomly selected a novel growth medium mixture from one of nine possible media mixtures for each population in each generation. Each population in the fluctuating environment therefore experienced a unique series of environmental conditions. The nine possible media mixtures represented a gradient of corn flour to natal medium ratios and were designed to yield expected population growth rates between 0.88 and 1.33 (Supporting information Table S1). We chose this range to mimic environmental stochasticity measured in nature (Sæther & Engen, 2002) while remaining within the bounds of biologically realistic population growth (λ between 0.5 and 1.5) (Morris et al., 2008). We chose random sequences of growth media such that the expected geometric mean population growth rate for the population experiencing that sequence resembled expected growth of populations in the stable environment (λ_expected_ = 1.2 ± 0.05).

We estimated the amount of environmental stochasticity that we achieved in the fluctuating environment as the difference in mean total stochasticity between populations in the fluctuating and stable environments (Sæther & Engen, 2002). We assumed that total stochasticity was a combination of demographic and environmental stochasticity for populations in the fluctuating environment, and that demographic stochasticity was the sole contributor to total stochasticity for populations in the stable environment. Total stochasticity (demographic plus environmental) of each population that did not experience extinction (*n* = 667) was calculated as the variance of the natural logarithms of its population growth rates through seven generations following Sæther and Engen (2002):

(10) $$s_{\mathrm{total}}\;=\;s_{\mathrm{demographic}}\;+\;s_{\mathrm{environmental}}\;=\;\text{var}\left(\log\left(\lambda_{t}\right)\right)$$

where, for a particular population, *s*_total_ is its total stochasticity, *s*_demographic_ is its demographic stochasticity, *s*_environmental_ is its environmental stochasticity (assumed to be 0 for populations in the stable environment), and λ_*t*_ is its per capita population growth rate between generation *t*−1 and generation *t* (*t =* 1, 2, …, 7). We only calculated total stochasticity for populations that did not experience any extinction in order to capture the full temporal extent of environmental fluctuations and because extinctions would have an infinite effect on this measure of stochasticity.

### 2.5 Statistical analyses

We evaluated how our environment treatment affected variability in population growth rate (total stochasticity from Equation (10)) using a linear mixed effects model with environment (stable or fluctuating) as a fixed effect and block as a random intercept effect.

We used a mixed effects logistic regression with a logit link to predict the binary response of establishment, and a mixed effects Poisson regression with a log link to analyze population size. In both models, introduction regime, environment treatment, and their interaction were treated as fixed effects, and block was treated as a random intercept effect.

We assessed the effect of temporary extinctions on the establishment probability and mean population size by fitting the generalized linear mixed effects models described above to data from the multiple introduction regimes (i.e., not the 20 × 1 regime) and with additional predictor variables. To assess the effect of the presence of a temporary extinction, we included an additional Boolean predictor for whether a population went temporarily extinct or not. To assess the effect of total propagule pressure, we used the number of beetles introduced after the latest temporary extinction as a predictor because only introductions after the latest temporary extinction contribute to total propagule pressure.

Group‐level significance of fixed effects was tested using likelihood ratio tests on nested models, and least‐squares contrasts were used to compare levels of the fixed effects. All statistical analyses were performed in R, version 3.3.2 (R Core Team 2017). Generalized linear mixed models were fit using the lme4 package, version 1.1‐12 (Bates, Machler, Bolker, & Walker, 2015) and pairwise comparisons were made using the lsmeans package, version 2.25 (Lenth, 2016).

### 2.6 Data availability

The raw experiment data, simulated population trajectories, R code for the simulations, R code fitting the NBBg model, samples from the posterior distributions of the NBBg parameters, and R code fitting the mixed effects models are available as a version controlled repository on Figshare (https://doi.org/10.6084/m9.figshare.4648865).

### 2.7 Note on egg contamination

Laboratory procedures after generation 3 resulted in occasional egg contamination between replicate populations of the same introduction regime/environmental variability treatment. To estimate the extent and magnitude of contamination, we examined trajectories of populations having only one individual and no additional introduction events, which should have deterministically gone extinct. Of these 49 populations, 12 did not go extinct (24.5%) and instead persisted at a small size (min = 1, max = 3, mean = 1.9). For colonization success analyses, we manually edited population trajectories such that they went extinct in the generation after having only one individual.

## 3 RESULTS

### 3.1 Simulations

Summary statistics for the posterior distributions of the four NBBg model parameters are given in Supporting information Table S2. Posterior and prior distributions are shown in Supporting information Figure S1.

**Figure 1:**
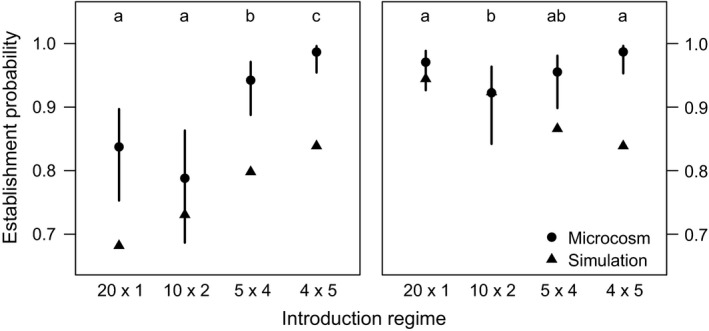
Establishment probabilities assessed seven generations after the first introduction event (*left panel*) and three generations after the final introduction event (*right panel*) for each introduction regime. Because different introduction regimes took different numbers of generations to complete, assessments made three generations after the final introduction event were made in different absolute experimental generations (*i.e.,* 20 × 1 assessment made at generation 3, 10 × 2 assessment made at generation 4, 4 × 5 assessment made at generation 6, and 5 × 4 assessment made at generation 7). Triangles represent results from simulations. Dot‐whiskers represent estimates and 95% confidence intervals from the mixed effects logistic regression model fit to data from the microcosm experiment. Different letters over dot‐whiskers represent introduction regime/environment combinations with significantly different establishment probabilities in the microcosm experiment

**Figure 2:**
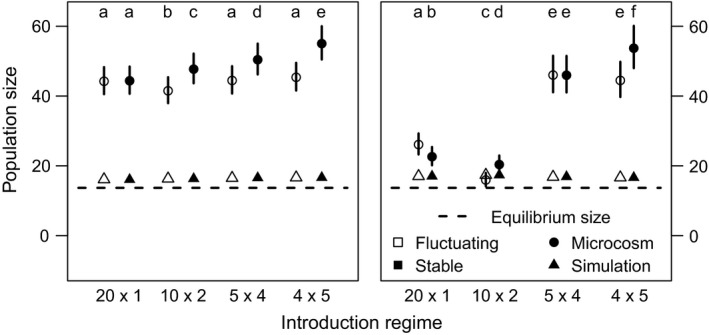
Mean sizes of extant populations across treatments assessed seven generations after the first introduction event (*left panel*) and three generations after the final introduction event (*right panel*) for each introduction regime. Because different introduction regimes took different numbers of generations to complete, assessments made three generations after the final introduction event were made in different absolute experimental generations (*i.e.,* 20 × 1 assessment made at generation 3, 10 × 2 assessment made at generation 4, 4 × 5 assessment made at generation 6, and 5 × 4 assessment made at generation 7). Triangles represent results from simulations. Dot‐whiskers represent estimates and 95% confidence intervals from the mixed effects Poisson regression model fit to data from the microcosm experiment. Different letters over dot‐whiskers represent introduction regimes with significantly different mean population sizes. The dashed line represents the theoretical equilibrium population size derived using Equation (2), which includes extinction and is therefore lower than the mean population size from the simulations

Our simulations showed introduction regimes with more introduction events were more likely to establish a population by the seventh generation (Figure 1). Introduction regimes with fewer introduction events were more likely to establish populations by three generations after the final introduction event. Simulated introductions into a stochastically fluctuating environment resulted in slightly lower population establishment for all introduction regimes (difference of ~1%), but did not favor any particular regime (not shown). The mean population sizes for each introduction regime/environment combination were approximately equal in simulations. Mean population sizes only incorporated extant populations, so they were slightly larger than the expectation for the equilibrium population size (Figure 2).

### 3.2 Microcosm experiment

Mean environmental stochasticity of populations in the fluctuating environment was 0.052 (95% CI = 0.0073 to 0.0966; *p* = 0.023). This value is near the median value of 0.055 measured in nature by Sæther and Engen (2002) in a meta‐analysis of 35 avian populations, indicating that we achieved biologically realistic fluctuations in population growth rate.

**Figure 3:**
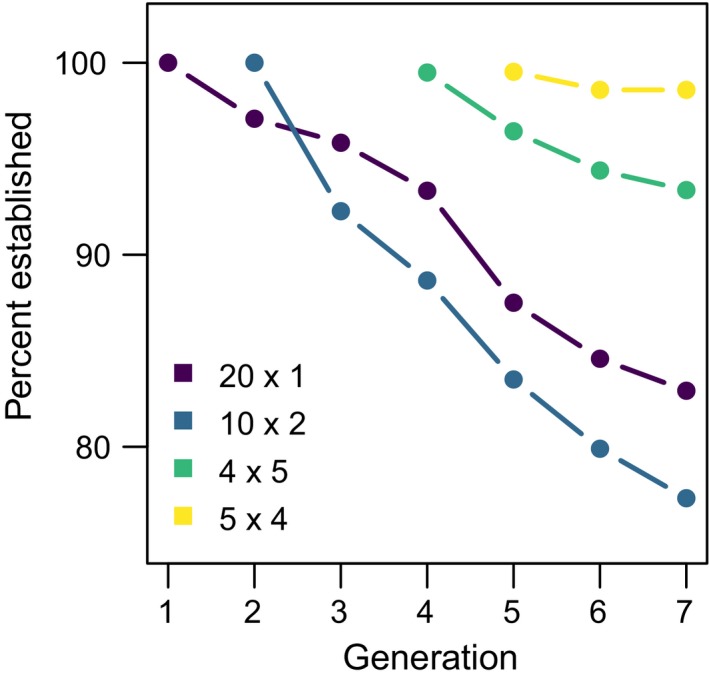
Percent of microcosm populations that were established in each generation for the four different introduction regimes. Data are pooled across the two environmental variability treatments. The introduction regimes are as follows: 20 × 1 = 20 individuals in the first generation, 10 × 2 = 10 individuals in each of the first two generations, 5 × 4 = 5 individuals in each of the first four generations, and 4 × 5 = 4 individuals in each of the first five generations

We found no evidence that the probability of establishment was affected by a main effect of environment (χ^2^ = 0.72, *df* = 1, *p* = 0.40 for generation 7 assessment; χ^2^ = 0.25, *df* = 1, *p* = 0.62 for assessment three generations after final introduction), nor by an interaction between environment and introduction regime when establishment was assessed at generation 7 (χ^2^ = 3.49, *df* = 3, *p* = 0.32). We detected a significant effect of an introduction regime/environment interaction when assessing establishment probability three generations after the final introduction event (χ^2^ = 16.61, *df* = 3, *p* = 0.0008). There was strong support for an effect of introduction regime on establishment probability (χ^2^ = 59.76, *df* = 3, *p* < 0.0001 for generation 7 assessment; χ^2^ = 17.52, *df* = 3, *p* = 0.0006 for assessment three generations after final introduction). Pairwise comparisons of the different introduction regimes averaged across the environment treatments revealed that the 4 × 5 regime was the most likely to establish populations by generation 7, with a probability of about 0.98, whereas the 20 × 1 and 10 × 2 regimes were the least likely to establish populations, with a probability reduced to about 0.8 (Figure 1 left panel, Figure 3). A similar pattern emerged for establishment three generations after the final introduction, with the 4 × 5 regime being the most likely to establish a population with a probability of 99% (although not statistically distinguishable from the 20 × 1 or 5 × 4 regimes) and the 10 × 2 regime being the least likely to establish a population with a probability reduced to 92% (Figure 1 right panel).

**Figure 4:**
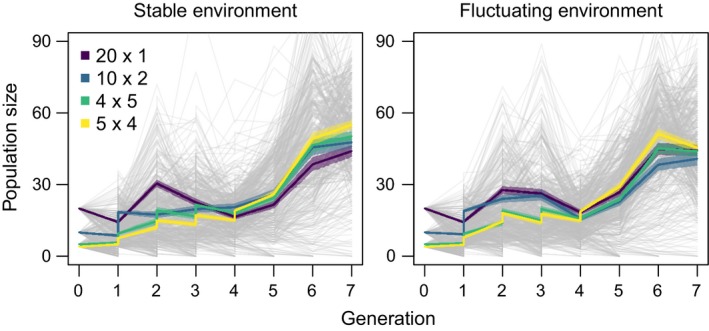
Population trajectories for 842 populations of *Tribolium* in the microcosm experiment. The first panel represents all populations in stable environments (*n* = 418) and the second panel represents all populations in fluctuating environments (*n* = 424). Vertical steps in the population size represent additional migrant individuals in introduction regimes with more than one introduction. Light grey lines represent individual population trajectories, and colored lines represent the mean size of extant populations for each of the four introduction regimes. The introduction regimes are as follows: 20 × 1 = 20 individuals in the first generation, 10 × 2 = 10 individuals in each of the first two generations, 5 × 4 = 5 individuals in each of the first four generations, and 4 × 5 = 4 individuals in each of the first five generations. Shading around the colored lines represents the mean size of extant populations plus and minus one standard error

The sizes of populations at generation 7 and 3 generations after their final introduction event were shaped by significant effects of introduction regime (χ^2^ = 91.65, *df* = 3, *p* < 0.0001 for population size at generation 7; χ^2^ = 134.83, *df* = 3, *p* < 0.0001 for population size 3 generations after final introduction), environment treatment (χ^2^ = 117.83, *df* = 3, *p* < 0.0001 for population size at generation 7; χ^2^ = 33.18, *df* = 3, *p* < 0.0001 for population size 3 generations after final introduction), and their interaction (χ^2^ = 44.62, *df* = 3, *p* < 0.0001 for population size at generation 7; χ^2^ = 4194.8, *df* = 3, *p* < 0.0001 for population size 3 generations after final introduction). For assessments made at generation 7 and 3 generations after the final introduction event, populations established via more introduction events were generally larger when averaged across the environment treatments. Extant populations in the stable environment were larger than those in the fluctuating environment when averaged across the introduction regimes. The interaction manifests as the benefit of a stable environment increases with more, smaller introduction events (Figures 2 and 4).

Extinctions accumulated regularly throughout the experiment period, with 101 of 842 populations (12.0%) going extinct by generation 7 (Figure 3). The additional introductions that some populations received often restored a population that had temporarily gone extinct. Of 602 populations that received more than one introduction (i.e., not the 20 × 1 introduction regime), 104 of them (17.3%) temporarily went extinct at least once before being replenished by additional colonizing individuals. Twelve populations were rescued in this way at least twice, and one population was rescued in this way three times.

Temporary extinctions significantly affected colonization success. The presence of a temporary extinction significantly reduced average establishment probability by generation 7 from 92.4% to 82.1% (difference = −1.13 on logit scale, 95% CI = −0.22 to −2.04, χ^2^ = 5.44, *df* = 1, *p* = 0.020) and mean population size from 47.8 to 45.4 (difference = −0.052 on log scale, 95% CI = −0.02 to −0.09, χ^2^ = 9.42, *df* = 1, *p* = 0.0021). Each additional colonist contributing to a population after the latest temporary extinction significantly increased the mean population size (estimate = 0.005 on the log scale, 95% CI = 6.4e‐05 to 0.01, χ^2^ = 3.94, *df* = 1, *p* = 0.047).

## 4 DISCUSSION

We assessed how the number and size of introduction events through time drive colonization success in a novel, harsh environment when the total number of individuals introduced to a location is fixed. We considered novel environments that were either stable or randomly fluctuating in quality through time and evaluated populations through seven discrete generations. We approached this question in two ways: (a) stochastic simulations of a demographic population dynamics model parameterized with empirical data, and (b) a highly replicated laboratory microcosm experiment. By coupling these approaches, we were able to test the theoretical understanding of how the introduction regime affects colonization in stable and fluctuating environments as well as develop new avenues for research when results from the two approaches did not align. We found that several small introductions increase colonization success and that demographic processes alone are insufficient to explain the dynamics observed in the experiment.

The rescue effect of multiple introductions can act demographically by increasing the size of populations (Hufbauer et al., 2015). Certainly, demographic rescue played a critical role for the 104 populations that went extinct temporarily until another introduction event revived them. Those temporary extinctions had lasting effects on colonization success. Colonization success declined for populations that experienced a temporary extinction, and the mean population size significantly increased if more colonists contributed to the population after a temporary extinction. These results reflect the overarching importance of total propagule pressure regardless of introduction regime.

We found minimal to no effect of a biologically realistic level of environmental stochasticity on establishment probability in demographic simulations at any assessment time point or in the microcosm when establishment was assessed at generation 7. Our results corroborate those of Cassey et al. (2014) who simulated introductions through time and also found a minimal effect of environmental stochasticity on establishment probability. Cassey et al. (2014) further found a minimal effect of random, infrequent catastrophes and bonanzas, suggesting that increasing temporal environmental variability in the broadest sense (i.e., encompassing environmental stochasticity, random catastrophes, and bonanzas) does not magnify the benefit of several, small introductions through time. A minimal role of environmental stochasticity contrasts with the results of Grevstad (1999) who found with simulations that several, small introductions would produce an especially high establishment probability compared to a single, large introduction in a variable environment. The difference in findings is perhaps due to a difference in the kinds of introduction regimes modeled: Grevstad (1999) simulated multiple introductions in space with strong environmental stochasticity often leading to catastrophic mortality, while we focused on introductions separated in time with less extreme environmental stochasticity leading to moderate fluctuations in growth rates (Lande, 1993).

Population dynamics in demographic simulations were likely to have been affected by compensatory density dependence. *Tribolium* beetles in challenging environments experience negative density dependence arising from egg cannibalism, which is more common in harsh environments (Via, 1999). From our NBBg Ricker model fit, we estimated the egg cannibalism rate, α, to be relatively high compared to previous estimates on the more benign natal growth medium (our estimated mean α was 0.0087 while the mean of our prior taken from Melbourne and Hastings (2008) was 0.0037; see Supporting information Figure S1). This high cannibalism rate was incorporated as a key dynamic in our simulations and may have overwhelmed any effect of environmental variability. Furthermore, the strong negative density dependence in the simulations likely led to the convergence of the mean population sizes by generation 7 and 3 generations after final introduction events across all treatments. A similar convergence in population size was not observed in the microcosm, but oscillating population sizes were observed for all treatments (Figure 4) suggesting some role for compensatory density dependence in the experiment as well.

Density dependence is likely to interact with introduction regime to affect colonization success. For instance, fewer larger introductions are more likely to establish populations when positive density dependence is present (Drolet & Locke, 2016; Grevstad, 1999). Wittmann, Metzler, Gabriel, and Jeschke (2014) further show an effect of per capita population growth in systems with negative density dependence: when population growth rate is consistently greater than one in those systems, population establishment is faster with several smaller introductions, but when population growth rate is mixed (sometimes greater than one and sometimes less than one depending on population size), population establishment occurs fastest with fewer larger introductions. Strong negative density dependence led to an expected equilibrium population size below the total propagule pressure in this experiment (13.7 individuals; Equation (2); Figure 2). Thus, our simulated populations experienced the “mixed” scenario described by Wittmann et al. (2014), and predictably benefitted from fewer, larger introductions when standardizing our assessment of establishment probability to three generations after the final introduction event.

Population growth rate may also interact with introduction regime to affect colonization success. Cassey et al. (2014) suggested that their lower simulated mean population growth rates, where *R* was between 1.0 and 1.38, compared to that of Grevstad (1999), where *R* was equal to 2.0, explained why they found that a single large introduction always led to a greater establishment probability, while Grevstad (1999) found several small introductions to be more successful. However, our expected mean population growth rate was relatively low (*R *=* *1.132; Supporting information Table S2) and we still found that several small introductions had the greatest colonization success at generation 7 (left panels of Figures 1 and 2). This may be partially explained by the recency of the final introduction for populations in different introduction regimes, which affected the number of opportunities for stochastic extinction. When standardizing the number of introductions since the final introduction event for each introduction regime, fewer larger introductions do increase establishment probability in simulations (Figure 1, right panel). However, even when standardizing the time since final introduction, several small introductions in the microcosm produced larger populations (Figure 2, right panel) and a similar pattern of establishment probability across introduction regimes compared to assessment at generation 7 (compare left and right panels of Figure 1). Thus, our experiment results contradict the notion that net reproductive rate was the key control on how introduction regime affected colonization success, which merits further investigation.

We observed striking differences in the measures of colonization success between the microcosm experiment and the demographic simulations. We found that establishment probability increased with the number of introduction events in both the experiment and the simulations after seven generations, but that all experiment establishment probabilities equaled or exceeded expectations from simulations. In the experiment, mean population size in stable environments was greater than in fluctuating environments, and there was an interaction between environment and introduction regime whereby the mean population size was increasingly greater in stable compared to fluctuating environments as the number of introductions increased. Furthermore, populations grew larger by generation 7 and 3 generations after the final introduction in the experiment compared to the simulations.

Differences between the results of the simulations and of the microcosm experiment suggest that the demographic processes captured by the model do not account for all of the biological processes that occurred in the microcosm. Alternatively, the biological processes captured by the independent experiment used to fit the NBBg Ricker model (the parameters of which were then used for simulations) may not represent the dynamics of the propagule pressure microcosm experiment. Recent work shows that adaptation to the novel, harsh environment from standing variation is possible in this species within a similar timeframe as our experiment (Hufbauer et al., 2015; Szűcs, Vahsen, et al., 2017), and likely explains the greater establishment probability and population sizes in the microcosm compared to expectations derived from demographic simulations which do not include adaptation.

The rescue effect of multiple introductions can also act genetically by increasing the fitness of populations (Frankham, 2015; Hufbauer et al., 2015; Whiteley, Fitzpatrick, ChrisFunk, & Tallmon, 2015). The experimental immigrants all came from the same source population, so it is unlikely that gene flow from migrants united previously separated alleles into high‐fitness genotypes (Novak, 2007). Thus, a more likely mechanism by which immigration increased mean population size beyond expectations was by relieving inbreeding depression or counteracting drift‐induced allele loss. Small populations are more prone to experiencing increased homozygosity and inbreeding depression, which can reduce population growth rates and increase extinction risk (McCauley & Wade, 1981; O'Grady et al., 2006; Szűcs, Melbourne, Tuff, Weiss‐Lehman, & Hufbauer, 2017). Even small amounts of gene flow can alleviate these effects, so the additional small introductions of mated individuals from the external source population were well‐suited to bring about longer‐term relief (Hufbauer et al., 2015; Slatkin, 1985). However, Cassey et al. (2014) found that simulated inbreeding depression was especially detrimental for several, small introductions through time, so other mechanisms are likely at play.

One such mechanism may be adaptation of the incipient population, which is affected by sustained immigration. Introductions to a harsh, novel habitat can result in adaptive evolution with the right amount of gene flow if additional immigrants to a declining population prevent extinction long enough to allow for adaptation to occur (Holt & Gomulkiewicz, 1997). The strong compensatory density dependence as a result of egg cannibalism may ultimately provide a pathway for adaptation, as cannibalism rates are genetically variable and can confer individual fitness advantage via reduced development time (Via, 1999). However, too much gene flow can lead to genetic swamping whereby the homogenizing effects of gene flow overpowers ongoing local adaptation (Lenormand, 2002). This may have been the case for populations in the 10 × 2 introduction regime, which had the highest average migration rate, lowest establishment probability, and lowest mean population size. Alternatively, negative density dependence may have reduced population fitness when migration rates were high, reducing population growth rates and hampering the spread of adaptive alleles (Holt & Gomulkiewicz, 1997). Although not significant, the lower establishment probability and mean population size in the 10 × 2 introduction regime warrants further work to assess whether they exemplify the yet‐unseen scenario described by Blackburn, Lockwood, and Cassey (2015) in which maladaptive gene flow from multiple introductions hampers population establishment in a novel range. More broadly, further study is necessary to evaluate how immigration affected adaptation in this system, if at all (Boulding & Hay, 2001; Gomulkiewicz & Holt, 1995).

## 5 CONCLUSION

Our experimental results suggest that several, small introductions through time lead to greater colonization success in a novel habitat, and that introductions into a stable recipient environment lead to larger population sizes, but not greater establishment probability. Furthermore, introductions to a stable recipient environment are especially beneficial to populations established with more introduction events. These results defied our expectations derived from parallel simulations of a model that included demographic processes but not evolutionary ones, so we suspect a genetic mechanism might be at work. Genetic mechanisms are rarely incorporated when simulating the effect of introduction regime on colonization (but see Cassey et al., 2014), and our multigeneration microcosm is unique in bringing evolutionary processes to bear on parsing two key components of propagule pressure in an experimental setting.

For invasions, our results highlight the importance; University of California Davis Libraries Open Access Fund; Colorado State University Libraries Open Access Research and Scholarship Fund of preventing further introductions to the same location, even for established species. For conservation and biological control, our results suggest that emphasis should be placed on increasing the number of introductions or reintroductions to a location, rather than increasing the size of those events if Allee effects are weak. Sustained introduction efforts should also bring about concomitant benefits in the form of longer‐term monitoring, increased data collection, and more opportunities for experimentation and adaptive management (Godefroid et al., 2011; Lockwood et al., 2005).

## CONFLICT OF INTEREST

None declared.

## AUTHOR CONTRIBUTIONS

MJK, RAH, and MFO designed the experiment. MJK, MFO, and RAH collected the data. MJK and BAM designed the simulations. MJK implemented the simulations. MJK, RAH, MFO, and BAM wrote the paper.

## References

[ece34226-bib-0001] Bates, D. , Machler, M. , Bolker, B. , & Walker, S. (2015). Fitting linear mixed‐effects models using lme4. Journal of Statistical Software, 67, 1–48.

[ece34226-bib-0002] Berggren, A. (2001). Colonization success in Roesel's bush‐cricket *Metrioptera roeseli*: The effects of propagule size. Ecology, 82, 274–280. 10.1890/0012-9658(2001)082[0274:CSIRSB]2.0.CO;2

[ece34226-bib-0003] Blackburn, T. M. , & Duncan, R. P. (2001). Establishment patterns of exotic birds are constrained by non‐random patterns in introduction. Journal of Biogeography, 28, 927–939.

[ece34226-bib-0004] Blackburn, T. M. , Lockwood, J. L. , & Cassey, P. (2015). The influence of numbers on invasion success. Molecular Ecology, 24, 1–12. 25641210 10.1111/mec.13075

[ece34226-bib-0005] Boulding, E. G. , & Hay, T. (2001). Genetic and demographic parameters determining population persistence after a discrete change in the environment. Heredity (Edinb), 86, 313–324. 10.1046/j.1365-2540.2001.00829.x 11488968

[ece34226-bib-0006] Cassey, P. , Blackburn, T. M. , Duncan, R. P. , & Chown, Sl. (2005). Concerning invasive species: Reply to Brown and Sax. Austral Ecology, 30, 475–480. 10.1111/j.1442-9993.2005.01505.x

[ece34226-bib-0007] Cassey, P. , Prowse, T. A. A. , & Blackburn, T. M. (2014). A population model for predicting the successful establishment of introduced bird species. Oecologia, 175, 417–428. 10.1007/s00442-014-2902-1 24566638

[ece34226-bib-0008] Colautti, R. I. , Grigorovich, I. A. , & MacIsaac, H. J. (2006). Propagule pressure: A null model for biological invasions. Biological Invasions, 8, 1023–1037. 10.1007/s10530-005-3735-y

[ece34226-bib-0009] de Valpine, P. , Turek, D. , Paciorek, C. J. , Anderson‐Bergman, C. , Temple Lang, D. , & Bodik, R. (2016). Programming with models: Writing statistical algorithms for general model structures with NIMBLE. Journal of Computational and Graphical Statistics, 8600, 1–20.

[ece34226-bib-0010] Drake, J. M. , Baggenstos, P. , & Lodge, D. M. (2005). Propagule pressure and persistence in experimental populations. Biology Letters, 1, 480–483. 10.1098/rsbl.2005.0375 17148238 PMC1626370

[ece34226-bib-0011] Drolet, D. , & Locke, A. (2016). Relative importance of propagule size and propagule number for establishment of non‐indigenous species: A stochastic simulation study. Aquatic Invasions, 11, 101–110. 10.3391/ai.2016.11.1.11

[ece34226-bib-0012] Fauvergue, X. , Vercken, E. , Malausa, T. , & Hufbauer, R. A. (2012). The biology of small, introduced populations, with special reference to biological control. Evolutionary Applications, 5, 424–443. 10.1111/j.1752-4571.2012.00272.x 22949919 PMC3407862

[ece34226-bib-0013] Frankham, R. (2015). Genetic rescue of small inbred populations: Meta‐analysis reveals large and consistent benefits of gene flow. Molecular Ecology, 24, 2610–2618. 10.1111/mec.13139 25740414

[ece34226-bib-0014] Godefroid, S. , Piazza, C. , Rossi, G. , Buord, S. , Stevens, A.‐D. , Aguraiuja, R. , … Vanderborght, T. (2011). How successful are plant species reintroductions? Biological Conservation, 144, 672–682. 10.1016/j.biocon.2010.10.003

[ece34226-bib-0015] Gomulkiewicz, R. , & Holt, R. D. (1995). When does evolution by natural selection prevent extinction? Evolution (N.Y.), 49, 201–207. 10.1111/j.1558-5646.1995.tb05971.x 28593677

[ece34226-bib-0016] Grevstad, F. S. (1999). Factors influencing the chance of population establishment: Implications for release strategies in biocontrol. Ecological Applications, 9, 1439–1447. 10.1890/1051-0761(1999)009[1439:FITCOP]2.0.CO;2

[ece34226-bib-0017] Grevstad, F. S. , Coombs, E. M. , & McEvoy, P. B. (2011). Revisiting release strategies in biological control of weeds: Are we using enough releases? In *Proceedings of the XIII international symposium on biological control of weeds*, Waikoloa, Hawai‘i, USA, September 11–16 (pp. 368–376).

[ece34226-bib-0018] Haccou, P. , & Iwasa, Y. (1996). Establishment probability in fluctuating environments: A branching process model. Theoretical Population Biology, 50, 254–280. 10.1006/tpbi.1996.0031 9000490

[ece34226-bib-0019] Haccou, P. , & Vatutin, V. (2003). Establishment success and extinction risk in autocorrelated environments. Theoretical Population Biology, 64, 303–314. 10.1016/S0040-5809(03)00092-3 14522171

[ece34226-bib-0020] Hedge, L. H. , O'Connor, W. A. , & Johnston, E. L. (2012). Manipulating the intrinsic parameters of propagule pressure: Implications for bio‐invasion. Ecosphere, 3, 1–13.

[ece34226-bib-0021] Holt, R. D. , & Gomulkiewicz, R. (1997). How does immigration influence local adaptation? A reexamination of a familiar paradigm. American Naturalist, 149, 563–572. 10.1086/286005

[ece34226-bib-0022] Hopper, K. R. , & Roush, R. T. (1993). Mate finding, dispersal, number released, and the success of biological control introductions. Ecological Entomology, 18, 321–331. 10.1111/j.1365-2311.1993.tb01108.x

[ece34226-bib-0023] Hufbauer, R. A. , Szűcs, M. , Kasyon, E. , Youngberg, C. , Koontz, M. J. , Richards, C. , … Melbourne, B. A. (2015). Three types of rescue can avert extinction in a changing environment. Proceedings of the National Academy of Sciences of the United States of America, 112, 10557–10562. 10.1073/pnas.1504732112 26240320 PMC4547288

[ece34226-bib-0024] Jeschke, J. M. (2014). General hypotheses in invasion ecology. Diversity and Distributions, 20, 1229–1234. 10.1111/ddi.12258

[ece34226-bib-0025] Lande, R. (1988). Genetics and demography in biological conservation. Science, 241, 1455–1460. 10.1126/science.3420403 3420403

[ece34226-bib-0026] Lande, R. (1993). Risks of population extinction from demographic and environmental stochasticity and random catastrophes. American Naturalist, 142, 911–927. 10.1086/285580 29519140

[ece34226-bib-0027] Lenormand, T. (2002). Gene flow and the limits to natural selection. Trends in Ecology & Evolution, 17, 183–189. 10.1016/S0169-5347(02)02497-7

[ece34226-bib-0028] Lenth, R. V. (2016). Least‐squares means: The R package lsmeans. Journal of Statistical Software, 69, 1–33.

[ece34226-bib-0029] Lockwood, J. L. , Cassey, P. , & Blackburn, T. (2005). The role of propagule pressure in explaining species invasions. Trends in Ecology & Evolution, 20, 223–228. 10.1016/j.tree.2005.02.004 16701373

[ece34226-bib-0030] Lodge, D. M. (1993). Biological invasions: Lessons for ecology. Trends in Ecology & Evolution, 8, 133–137. 10.1016/0169-5347(93)90025-K 21236129

[ece34226-bib-0031] Mack, R. N. , Simberloff, D. , Lonsdale, W. M. , Evans, H. , Clout, M. , & Bazzaz, F. A. (2000). Biotic invasions: Causes, epidemiology, global consequences, and control. Ecological Applications, 10, 689–710. 10.1890/1051-0761(2000)010[0689:BICEGC]2.0.CO;2

[ece34226-bib-0032] McCauley, D. E. , & Wade, M. J. (1981). The populational effects of inbreeding in *Tribolium* . Heredity (Edinb), 46, 59–67. 10.1038/hdy.1981.6

[ece34226-bib-0033] Melbourne, B. A. , & Hastings, A. (2008). Extinction risk depends strongly on factors contributing to stochasticity. Nature, 454, 100–103. 10.1038/nature06922 18596809

[ece34226-bib-0034] Memmott, J. , Craze, P. G. , Harman, H. M. , Syrett, P. , & Fowler, S. V. (2005). The effect of propagule size on the invasion of an alien insect. Journal of Animal Ecology, 74, 50–62.

[ece34226-bib-0035] Morris, W. F. , Pfister, C. A. , Tuljapurkar, S. , Haridas, C. V. , Boggs, C. L. , Boyce, M. S. , … Menges, E. S. (2008). Longevity can buffer plant and animal populations against changing climatic variability. Ecology, 89, 19–25. 10.1890/07-0774.1 18376542

[ece34226-bib-0036] Novak, S. J. (2007). The role of evolution in the invasion process. Proceedings of the National Academy of Sciences of the United States of America, 104, 3671–3672. 10.1073/pnas.0700224104 17360409 PMC1820639

[ece34226-bib-0037] O'Grady, J. J. , Brook, B. W. , Reed, D. H. , Ballou, J. D. , Tonkyn, D. W. , & Frankham, R. (2006). Realistic levels of inbreeding depression strongly affect extinction risk in wild populations. Biological Conservation, 133, 42–51. 10.1016/j.biocon.2006.05.016

[ece34226-bib-0038] R Core Team (2017). R: A language and environment for statistical computing. Retrieved from http://www.r-project.org/.

[ece34226-bib-0039] Ricciardi, A. (2007). Are modern biological invasions an unprecedented form of global change? Conservation Biology, 21, 329–336. 10.1111/j.1523-1739.2006.00615.x 17391183

[ece34226-bib-0040] Sæther, B.‐E. , & Engen, S. (2002). Pattern of variation in avian population growth rates. Philosophical Transactions of the Royal Society of London. Series B, Biological sciences, 357, 1185–1195. 10.1098/rstb.2002.1119 12396511 PMC1693028

[ece34226-bib-0041] Sakai, A. K. , Allendorf, F. W. , Holt, J. S. , Lodge, D. M. , Molofsky, J. , With, K. A. , … Weller, S. G. (2001). The population biology of invasive species. Annual Review of Ecology and Systematics, 32, 305–332. 10.1146/annurev.ecolsys.32.081501.114037

[ece34226-bib-0042] Schreiber, S. J. , & Lloyd‐Smith, J. O. (2009). Invasion dynamics in spatially heterogeneous environments. American Naturalist, 174, 490–505. 10.1086/605405 19737109

[ece34226-bib-0043] Simberloff, D. (1989). Which insect introductions succeed and which fail? In Drake J. A., Mooney H. A., di Castri F., Groves R. H., Kruger F. J., Rejmánek M. & Williamson M. (Eds.), Biological invasions: A global perspective (pp. 61–75). New York, New York, USA: John Wiley & Sons Ltd.

[ece34226-bib-0044] Simberloff, D. (2009). The role of propagule pressure in biological invasions. Annual Review of Ecology Evolution and Systematics, 40, 81–102. 10.1146/annurev.ecolsys.110308.120304

[ece34226-bib-0045] Sinclair, J. S. , & Arnott, S. E. (2016). Strength in size not numbers: Propagule size more important than number in sexually reproducing populations. Biological Invasions, 18, 497–505. 10.1007/s10530-015-1022-0

[ece34226-bib-0046] Slatkin, M. (1985). Gene flow in natural populations. Annual Review of Ecology and Systematics, 16, 393–430. 10.1146/annurev.es.16.110185.002141

[ece34226-bib-0047] Szűcs, M. , Melbourne, B. A. , Tuff, T. , Weiss‐Lehman, C. , & Hufbauer, R. A. (2017). Genetic and demographic founder effects have long‐term fitness consequences for colonising populations. Ecology Letters, 20, 1–7. 10.1111/ele.12743 28145080

[ece34226-bib-0048] Szűcs, M. , Vahsen, M. L. , Melbourne, B. A. , Hoover, C. , Weiss‐Lehman, C. , & Hufbauer, R. A. (2017). Rapid adaptive evolution in novel environments acts as an architect of population range expansion. Proceedings of the National Academy of Sciences of the United States of America, 114, 201712934. 10.1073/pnas.1712934114 PMC5754790 29183976

[ece34226-bib-0049] Taylor, S. S. , Jamieson, I. G. , & Armstrong, D. P. (2005). Successful island reintroductions of New Zealand robins and saddlebacks with small numbers of founders. Animal Conservation, 8, 415–420. 10.1017/S1367943005002337

[ece34226-bib-0050] Van Allen, B. G. , & Rudolf, V. H. W. (2013). Ghosts of habitats past: Environmental carry‐over effects drive population dynamics in novel habitat. American Naturalist, 181, 596–608. 10.1086/670127 23594544

[ece34226-bib-0051] Via, S. (1999). Cannibalism facilitates the use of a novel environment in the flour beetle, *Tribolium castaneum* . Heredity (Edinb), 82, 267–275. 10.1038/sj.hdy.6884820 10341438

[ece34226-bib-0052] Whiteley, A. R. , Fitzpatrick, S. W. , Chris Funk, W. , & Tallmon, D. A. (2015). Genetic rescue to the rescue. Trends in Ecology & Evolution, 30, 42–49. 10.1016/j.tree.2014.10.009 25435267

[ece34226-bib-0053] Wittmann, M. J. , Metzler, D. , Gabriel, W. , & Jeschke, J. M. (2014). Decomposing propagule pressure: The effects of propagule size and propagule frequency on invasion success. Oikos, 123, 441–450. 10.1111/j.1600-0706.2013.01025.x

[ece34226-bib-0054] Zenni, R. D. , & Nuñez, M. A. (2013). The elephant in the room: The role of failed invasions in understanding invasion biology. Oikos, 122, 801–815.

