# Supplementary material for: Parsing propagule pressure: Number, not size, of introductions drives colonization success in a novel environment
Source: Ecol Evol. 2018 Jul 20;8(16):8043–54. doi: 10.1002/ece3.4226 (PMC6145030; doi:10.1002/ece3.4226)
Supplement: Supplementary file 1 [file ECE3-8-8043-s001.pdf]

Supplementary Table 1. Novel growth medium mixtures and $\lambda_{expected}$ for populations in the fluctuating treatment. Mixture 5, with 0.95% natal medium and 99.05% corn flour, (bold) was used in each generation for populations in the stable treatment. $\lambda_{expected}$ was estimated from pilot studies using populations independent from the propagule pressure experiment (data not shown).

Mixture	% Natal	% Corn	$\lambda_{expected}$
1	0.580	99.420	0.877
2	0.72	99.328	0.981
3	0.765	99.235	1.070
4	0.857	99.143	1.141
**5**	**0.950**	**99.050**	**1.20**
6	1.042	98.958	1.245
7	1.135	98.865	1.282
8	1.228	98.772	1.311
9	1.320	98.680	1.333
natal	100	0	3.36

Supplementary Table 2. Estimates of key parameters from the NBBg Bayesian hierarchical model fit to population size data from 125 populations reared using the same growth medium mixture (mixture 5; Supplementary Table 1) and censused at time *t* and time *t+1*. The environmental stochasticity parameter for stable environments, $k_{E(stable)}$, was directly estimated by fitting the NBBg model to data from the 125 populations. The environmental stochasticity parameter for fluctuating environments, $k_{E(fluctuating)}$ was derived by multiplying the $k_{E(stable)}$ parameter distribution by 100/121, which results in a 10% increase in the standard deviation of the density independent population growth rate. A full text file of the burned-in samples that comprise the posterior distributions of these parameters is available. Note that a smaller k_E value corresponds to a greater environmental stochasticity because it is the shape parameter of the gamma distribution. Similarly, small values of k_D correspond to a greater demographic heterogeneity.

	Parameter mean	Standard deviation
R_0	1.132	0.0631
α	0.00874	0.00073
$k_{E\ (stable)}$	19.742	3.863
$k_{E\ (fluctuating)}$	16.316	3.192
k_D	2.250	1.021

Supplementary Table 3. Number of experimental replicates for each combination of introduction regime and environmental variability. Uneven sampling design is a consequence of an unexpected shortage of colonists during some temporal block/generation combinations.

		Introduction regime			
		20x1	10x2	5x4	4x5
Environmental variability	Stable	120	98	95	105
	Fluctuating	120	96	101	107

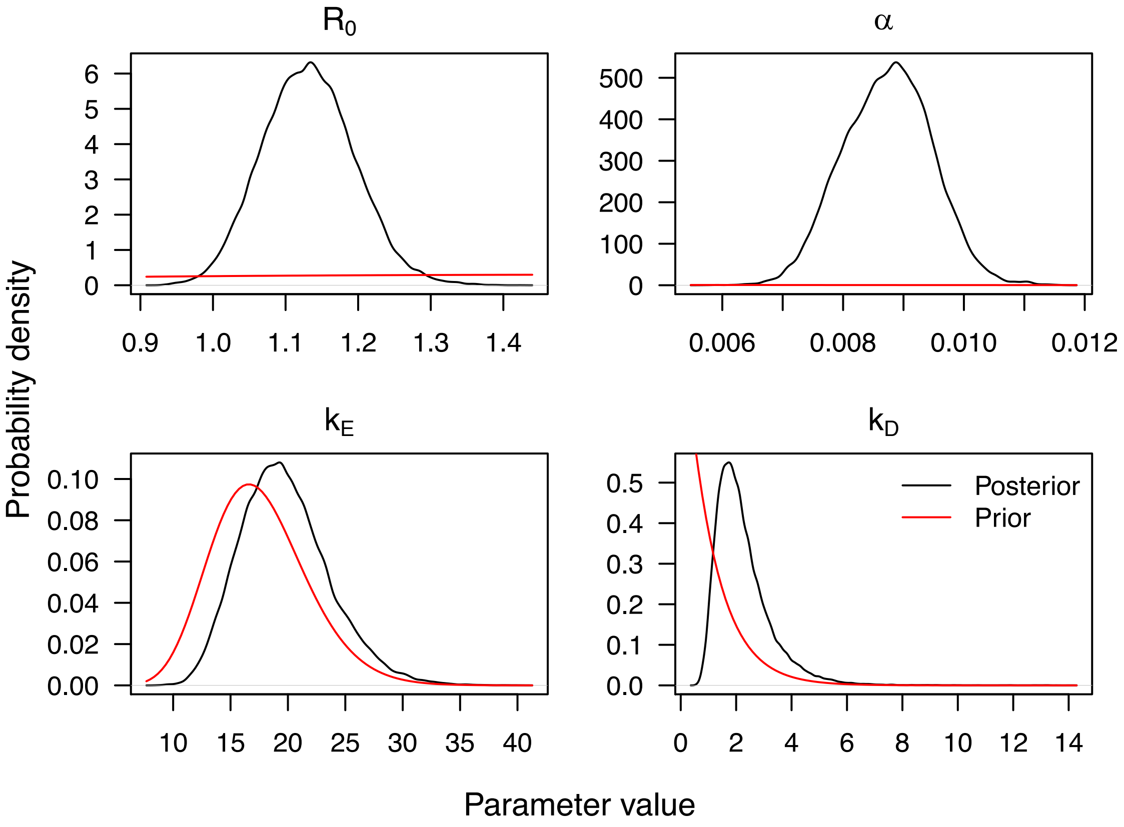

Supplementary Figure 1. Posterior and prior distributions for 4 key parameters estimated by fitting the NBBg model to 1 generation of census data for 125 populations. The prior for each parameter is gamma distributed with equal mean and variance (R_0 = 2.6, α = 0.0037, k_E = 17.6, k_D = 1.07). Prior means come from parameter estimates by Melbourne and Hastings (2008). The k_E posterior is similar to the prior because, though populations were reared using different growth media in our study (mixture 5; Supp. Table 1) versus that of Melbourne and Hastings (2008) (natal mixture; Supp Table 1), the same growth medium was used for all populations within each study. Thus, both estimates of k_E reflect the amount of environmental stochasticity in a controlled lab environment.

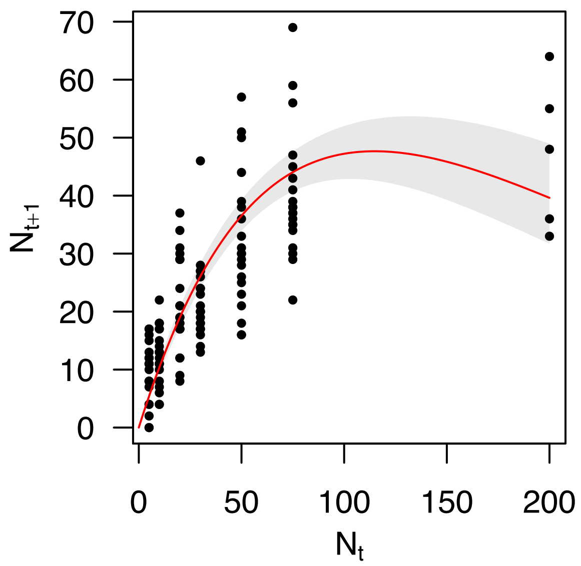

Supplementary Figure 2. Data used to fit the NBBg Ricker model from 125 populations of *Tribolium* flour beetles independent of the populations used in the propagule pressure microcosm experiment. Populations were founded at different densities (depicted on the x-axis) and censused one generation later (y-axis) following the microcosm protocol in the *Methods.* All populations were reared on the same novel growth medium (mixture 5; Supplementary Table 1). The red line depicts the expectation of the Ricker function using the mean values of R_0 and α of their posterior distributions. The shaded area represents the 95% credible interval around the expectation of the function.

Supplemental Methods

We implemented an effect of environmental stochasticity on density independent per capita growth rate in simulations via the shape parameter, k_E, for the gamma distribution (see Eq. 3 in the main text). Populations in both the stable environment and the fluctuating environment were influenced by environmental stochasticity, but the variance of the gamma distribution and thus the magnitude of the effect of environmental stochasticity was greater for the fluctuating environment though they had the same mean (Supplementary Equation 1).

$$E\left(R_{t,i}\right)_{stable} = E\left(R_{t,i}\right)_{fluctuating} = R_0 \qquad \text{Supplementary Equation 1}$$

The posterior distribution for k_E of the stable environment was estimated by fitting the NBBg Ricker model to census data across one generation for 125 populations reared at different initial population sizes (between 5 and 200 individuals; see Eq. 9 and description in main text as well as Supplementary Figure 2). The deterministic and stochastic components of the model are described in detail in the *Methods* section of the main text (Eq. 1 through 8). Fitting the NBBg Ricker model also generated the posterior distribution of the mean density independent per capita growth rate, R_0, which was used for all simulated populations. The mean and variance of the gamma distribution representing environmental stochasticity for the stable environment are given in Supplementary Equation 2:

$$Var(R_{t,i})_{stable} = \frac{{R_0}^2}{k_{E(stable)}}$$

$$Stdev(R_{t,i})_{stable} = \frac{R_0}{\sqrt{k_{E(stable)}}}$$ Supplementary Equation 2

To generate the posterior distribution of the k_E parameter for the fluctuating environment, we multiplied each of the samples from the posterior distribution of the k_E parameter for the stable environment by 100/121 (10^2 / 11^2) in order to increase the standard deviation of the environmental stochasticity gamma distribution for fluctuating environments by 10%. The steps taken to derive this increase in the standard deviation of the gamma distribution for the fluctuating environment are given in Supplementary Equation 3:

$$Var(R_{t,i})_{fluctuating} = \frac{{R_0}^2}{(\frac{10^2}{11^2})k_{E(stable)}}$$

$$Var(R_{t,i})_{fluctuating} = \frac{(11R_0)^2}{(10^2)k_{E(stable)}}$$

$$Stdev(R_{t,i})_{fluctuating} = \frac{11R_0}{10\sqrt{k_{E(stable)}}}$$

$$Stdev(R_{t,i})_{fluctuating} = \frac{11}{10}Stdev(R_{t,i})_{stable}$$ Supplementary Equation 3
